# Real-world outcome of immune checkpoint inhibitors for advanced hepatocellular carcinoma with macrovascular tumor thrombosis

**DOI:** 10.1007/s00262-020-02845-9

**Published:** 2021-01-06

**Authors:** Hong-Ming Tsai, Meng-Zhi Han, Yih-Jyh Lin, Ting-Tsung Chang, Chiung-Yu Chen, Pin-Nan Cheng, Chiao-Hsiung Chuang, I-Chin Wu, Po-Jun Chen, Jui-Wen Kang, Yen-Cheng Chiu, Hung-Chih Chiu, Shih-Chieh Chien, Hsin-Yu Kuo

**Affiliations:** 1 Department of Diagnostic Radiology, National Cheng Kung University Hospital, College of Medicine, National Cheng Kung University, Tainan, Taiwan; 2 Department of Internal Medicine, An Nan Hospital, China Medical University, Tainan, Taiwan; 3 Department of Surgery, National Cheng Kung University Hospital, College of Medicine, National Cheng Kung University, Tainan, Taiwan; 4 Department of Internal Medicine, National Cheng Kung University Hospital, College of Medicine, National Cheng Kung University, 138 Sheng Li Road, Tainan, Taiwan; 5 Institute of Clinical Medicine, College of Medicine, National Cheng Kung University, Tainan, Taiwan

**Keywords:** Hepatocellular carcinoma, Macrovascular invasion, Immunotherapy, Immune checkpoint inhibitors

## Abstract

Programmed cell death protein-1 (PD-1) inhibitors have shown promising results for treating advanced hepatocellular carcinoma (HCC). However, the clinical utility of such inhibitors in HCC patients with vascular tumor thrombosis remains unclear. This study investigated PD-1 inhibitor efficacy in advanced HCC with macrovascular invasion in a clinical setting. Among the 110 patients with unresectable HCC treated with PD-1 inhibitors, 34 patients with vascular metastases in the portal vein and inferior vena cava were retrospectively compared with 34 patients without tumor thrombi. The vascular response and its effect on survival were assessed. Predictors of survival were identified using multivariate analysis. Among patients achieving objective response, those with and without thrombi exhibited similar response to immunotherapy and comparable survival. Among the 34 patients with tumor thrombi, including 13 receiving PD-1 inhibitors alone and 21 receiving it in combination with tyrosine kinase inhibitors, the median overall survival was 8.9 months (95% confidence interval 3.2–12.6). The objective response rate of vascular metastasis was 52.9%, and vascular responders had a significantly longer survival than did non-responders (11.1 vs 3.9 months). Failure to obtain a vascular response correlated significantly with increased post-treatment Child–Pugh score or class. Multivariate analysis showed that vascular response was a significant positive factor for longer overall survival. Treatment-related grade 3/4 adverse events occurred in 3 (8.8%) of the patients with tumor thrombi. Immunotherapy with PD-1 inhibitors may be a feasible treatment option for HCC with tumor thrombi owing to the high response rate of tumor thrombi and favorable survival outcomes.

**Supplementary Information:**

The online version contains supplementary material available at 10.1007/s00262-020-02845-9.

## Introduction

Hepatocellular carcinoma (HCC) is the most common primary liver cancer and the fourth most common cause of cancer-related death worldwide [1]. Most HCC patients are diagnosed at an advanced stage, with 10–40% involving macrovascular invasion (MVI) [2]. HCC patients with MVI are not amenable to curative therapies and exhibit a very poor prognosis [3]. In the patients receiving no treatment, the median survival time is only 3 months. The few therapeutic modalities available to these patients have unsatisfactory survival benefits. The AASLD guidelines recommend sorafenib as the first-line systemic therapy for advanced HCC with MVI [4]. However, sorafenib has exhibited disappointing efficacy for the treatment of vascular invasion, with a disease control rate (DCR) of 33.3%, progression-free survival (PFS) of 2.0 months, and overall survival (OS) of 3.1 months [5, 6]. In addition, the incidence of treatment-related adverse events is as high as 80% in sorafenib-treated subjects [5].

Although studies have attempted to elucidate the oncogenic drivers of HCC, the therapeutic clinical applications derived from this molecular knowledge are relatively limited [7]. Immunotherapy using immune checkpoint inhibitors (ICIs) has shown its promising antitumor efficacy in certain cancer types, particularly in lung cancer and melanoma [8]. The immunogenicity of the HCC tumor microenvironment reportedly suggests that immunotherapy may be an efficacious therapeutic approach to treat HCC [9]. Immunotherapy using the checkpoint inhibitor of the programmed cell death protein-1 (PD-1) has been approved as a second-line treatment option for patients with advanced HCC [10]. The phase 1/2 CheckMate 040 and phase 2 keynote-224 studies have reported an objective response rate (ORR) of 14% and a median OS of 12 months for the advanced HCC patients treated with PD-1 inhibitors [11, 12]. The adverse events were reported as manageable, with the most common symptoms being fatigue, rash, and diarrhea.

Despite these promising results, concrete data regarding ICI treatment for advanced HCC patients with MVI in clinical settings are still rare [13–15]. Additionally, there have been no randomized clinical trials yet to assess the treatment outcomes with regard to these patients. This study assesses the efficacy of PD-1 inhibitor therapy for patients with advanced HCC and portal vein tumor thrombus (PVTT) or inferior vena cava thrombus (IVCT) in an actual clinical setting. In contrast to clinical trials that were conducted to investigate ICI use in advanced HCC, the present study cohort with advanced MVI patients reflects the actual clinical severity of advanced HCC encountered outside of the clinical trials.

## Materials and methods

### Patients

**Fig. 1:**
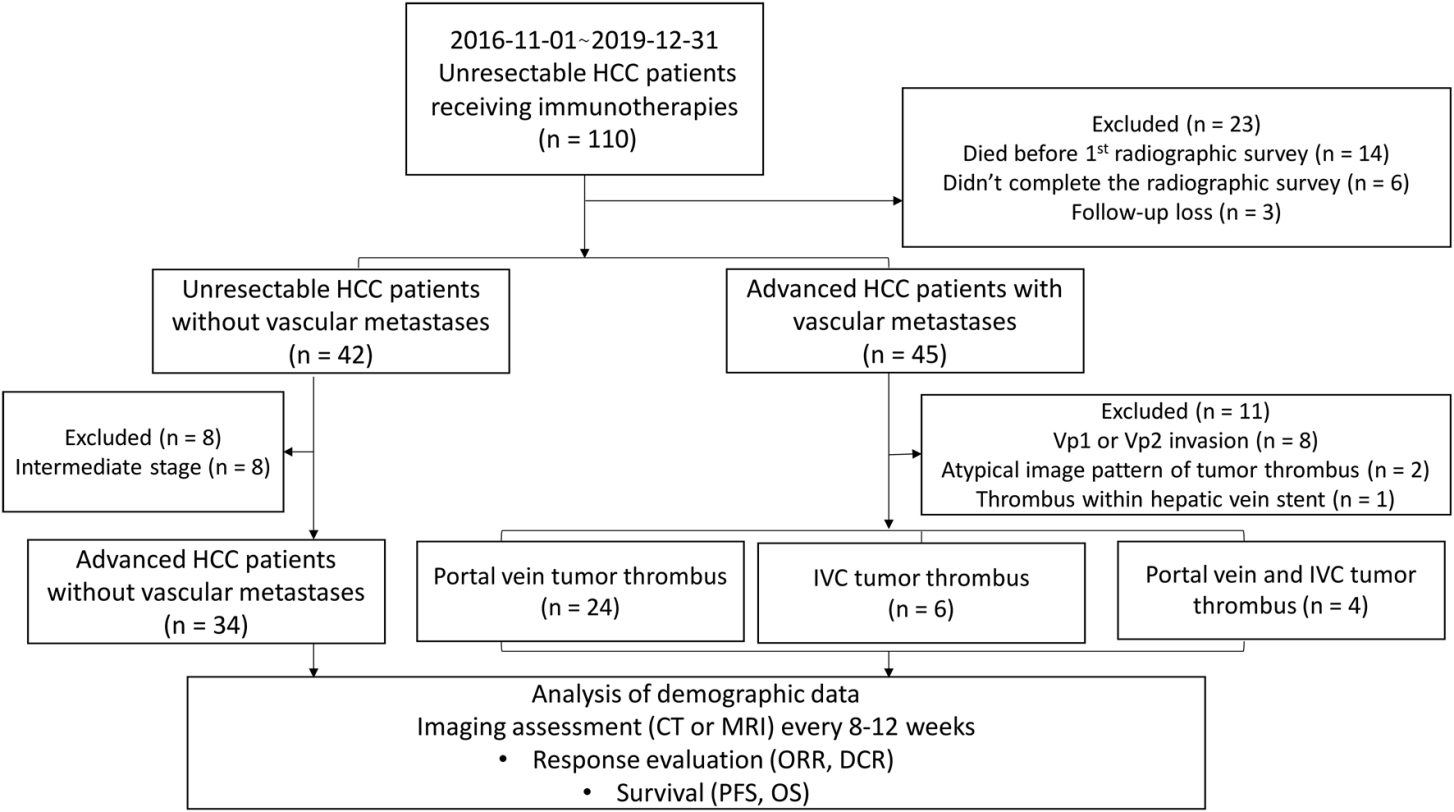
Study algorithm for subject selection. *HCC* hepatocellular carcinoma, *IVC* inferior vena cava vein, *CT* computed tomography, *MRI* magnetic resonance imaging, *ORR* overall response rate, *DCR* disease control rate, *PFS* progression-free survival, *OS* overall survival

In the period between November 1, 2016 and December 31, 2019, 110 patients with unresectable HCC were treated with PD‐1‐targeted immunotherapy using nivolumab or pembrolizumab at the National Cheng Kung University Hospital, Tainan, Taiwan. PD-1 inhibitors were prescribed to patients with advanced HCC who had no history of systemic therapy or progression after previous systemic regimens and to those with intermediate-stage HCC who experienced ineffective transarterial chemoembolization. We only included patients with advanced HCC who were subsequently assessed by radiological imaging for tumor response. Among the 110 patients, 23 were excluded due to incomplete planned radiographic evaluation (*n* = 6), death before first assessment with radiological imaging (*n* = 14), and failure to complete the treatment regimen (*n* = 3). Of the remaining 87 patients, 19 were excluded due to the presence of PVTT distal to or in the second-order branches of the portal vein (*n* = 8), equivocal imaging characteristics of tumor thrombosis (*n* = 3), and intermediate-stage HCC (*n* = 8). The remaining 68 patients, comprising those with major vascular invasion (main or first-branch PVTT and IVCT, *n* = 34) and those without vascular metastases (*n* = 34), met the study criteria and were included in the retrospective analysis (Fig. 1). The follow-up cut‐off date was set on February 28, 2020.

HCC diagnosis was based on tissue histology or typical radiographic findings [4]. The presence and extent of vascular invasion were diagnosed by characteristic findings using multiphase dynamic computed tomography (CT) or magnetic resonance imaging (MRI) [16, 17]. Malignant tumor thrombus was defined as thrombus enhancement after the administration of contrast media compared to pre-contrast images (≥ 20 HU on CT and ≥ 15% on MRI), thrombus expansion within the involved vessel, and continuity of thrombus within the tumor [18].

This study was approved by the Institutional Review Board of the National Cheng Kung University Hospital (AER-109-199) and performed in accordance with the ethical principles for medical research of the World Medical Association Declaration of Helsinki.

### Treatment and response evaluation

Patients received the standard dose of 3 mg/kg of intravenous nivolumab biweekly or 200 mg of intravenous pembrolizumab every 3 weeks. Adverse events were assessed using the National Cancer Institute Common Terminology Criteria for Adverse Events (NCI CTCAE; version 5.0).

The tumor response was evaluated using the Response Evaluation Criteria in Solid Tumors (RECIST) version 1.1 and modified RECIST (mRECIST) based on serial contrast-enhanced CT or MRI [19, 20].

To assess the vascular response, the largest diameters of the tumor thrombus were measured and compared with the basal value recorded [21] and categorized as follows: complete remission (CR), i.e., complete disappearance of the tumor thrombus; partial response (PR), i.e., at least a 30% decrease in thrombus diameters; stable disease (SD), i.e., a decrease of < 30% or an increase of < 20%; and progressive disease (PD), i.e., an increase of ≥ 20% in the sum of the diameters [13]. Objective response rate of tumor thrombi (ORRT) was defined as the total number of patients achieving CR or PR, and disease control rate of tumor thrombi (DCRT) was defined as the total number of patients achieving CR, PR, or SD. Patients achieving CR or PR were defined as responders, whereas patients achieving SD or PD were defined as non-responders. In the cases involving concurrent PVTT and IVCT, the vascular responses of the PVTT and IVCT were assessed individually.

### Statistical analyses

The Chi‐square test was used to compare categorical variables, and the unpaired Student's *t *test or Wilcoxon rank sum test was used to assess continuous variables. Survival curves were estimated using the Kaplan–Meier method and compared using the log-rank test. The OS was calculated from the date of PD-1 inhibitor commencement until death. PFS was calculated for the interval between treatment commencement and tumor progression according to RECIST or death from any cause, whichever came first. The univariate and multivariate analyses were performed using a Cox proportional hazards model to identify prognostic factors for survival. Statistically significant variables (*p* < 0.05) in the univariate analysis were chosen for inclusion in the multivariate analysis. A *p* value of < 0.05 was considered statistically significant. All analyses were conducted using the SAS statistical package (v. 9.4 for Windows; SAS Institute, Cary, NC, USA).

## Results

### Characteristics of patients

Overall, the baseline characteristics were balanced between the patients with and without tumor thrombi (Supplementary Table 1).

Among these patients with MVI, the presence of inferior vena cava (IVC) tumor thrombus was observed in 6 (17.6%), concurrent IVC and portal vein involvement in 4 (11.8%), main portal vein invasion in 16 (47.1%), and portal vein invasion at the first order branch in 8 (23.5%). On enrollment, 22 patients (64.7%) were diagnosed as Child–Pugh class A. Additionally, 21 patients (61.8%) were sorafenib-experienced, while 11 (32.4%) received PD-1 inhibitors as first-line systemic therapy. Combination therapy of PD-1 inhibitor and tyrosine kinase inhibitor (TKI) was administered to 21 patients (61.8%).

### Overall treatment response

The overall ORR of patients with and without tumor thrombi were 17.6% and 11.8% (*p* = 0.732), respectively, according to the RECIST and 20.6% and 17.7% (*p* = 1.000), respectively, according to the mRECIST.

Of the 34 patients with tumor thrombi assessed for overall tumor response, the ORR for Child–Pugh score class A and B were 27.3% and 0%, and the DCR were 40.9% vs 50.0%, respectively (Table 1). The ORR for ICI use as the first-line was 18.2%, and it was 17.3% for its use as the second- or third-line treatment (*p* = 1.000).

**Table 1:** Treatment response to immunotherapy in patients with macrovascular invasion

	Overall (*n* = 34)		Child–Pugh^a^ (*n* = 34)		Systemic ICI^a^ (*n* = 34)		Vessel (*n* = 34)	PVTT^b^ (*n* = 28)	IVCT^b^ (*n* = 10)
Response	mRECIST *n* (%)	RECIST *n* (%)	*A* (*n* = 22)	*B* (*n* = 12)	1st line (*n* = 11)	≥ 2nd line (*n* = 23)	*n* (%)	*n* (%)	*n* (%)
CR	1 (2.9)	0 (0)	0 (0)	0 (0)	0 (0)	0 (0)	2 (5.9)	2 (7.1)	0 (0)
PR	6 (17.6)	6 (17.6)	6 (27.3)	0 (0)	2 (18.2)	4 (17.4)	16 (47.1)	12 (42.9)	7 (70)
SD	7 (20.6)	9 (26.5)	3 (13.6)	6 (50)	3 (27.3)	6 (26.1)	4 (11.8)	4 (14.3)	0 (0)
PD	20 (58.8)	19 (55.9)	13 (59.1)	6 (50)	6 (54.5)	13 (56.5)	12 (35.3)	10 (35.7)	3 (30)
ORR	7 (20.6)	6 (17.6)	6 (27.3)	0 (0)	2 (18.2)	4 (17.3)	18 (52.9)	14 (50)	7 (70)
DCR	14 (41.2)	15 (44.1)	9 (40.9)	6 (50.0)	5 (45.5)	10 (43.5)	22 (64.7)	18 (64.3)	7 (70)

*CR* complete response, *PR* partial response, *SD* stable disease, *PD* progressive disease, *ORR* objective response rate, *DCR* disease control rate, *ICI* immune checkpoint inhibitor, *PVTT* portal vein tumor thrombus, *IVCT* inferior vena cava vein tumor thrombus, *RECIST* response evaluation criteria in solid tumors, *mRECIST* modified response evaluation criteria in solid tumors

^a^Response was evaluated using the RECIST

^b^For four patients with both PVTT and IVCT, vascular responses of the PVTT and IVCT were assessed individually

### Vascular tumor thrombus treatment response

As depicted in Table 1, of the 34 patients evaluated for vascular thrombus response, 2 (5.9%), 16 (47.1%), 4 (11.8%), and 12 (35.3%) patients achieved CR, PR, SD, and PD, respectively, resulting in an ORRT of 52.9% and a DCRT of 64.7%. The comparison of response rates between the PVTT and IVCT revealed ORRTs of 50% and 70% (*p* = 0.460) and DCRTs of 64.3% and 70% (*p* = 1.000), respectively.

Additionally, a significant increase in Child–Pugh scores and/or class post-treatment were observed for the vascular non-responders (SD or PD) when compared to the vascular responders (CR or PR) (75.0% vs 33.3%; *p* = 0.020), according to Table 2 and Supplementary Fig. 1. The rate of occurrence of new distant metastasis for the patients with non-responsive vascular thrombi was higher than those with a vascular response, but such difference was not quite significant (25% vs 5.6%; *p* = 0.164).

**Table 2:** Analysis of factors associated with non-responsiveness of vascular metastasis

	With tumor thrombi (*n* = 34)		*p*
	Vascular responders^a^ (*n* = 18)	Vascular non-responders^a^ (*n* = 16)	
Child–Pugh score/class elevation	6 (33.3)	12 (75.0)	0.020
New distant metastasis	1 (5.6)	4 (25.0)	0.164
Death	11 (61.1)	15 (93.8)	0.043
Ongoing ICI treatment	3 (16.7)	0 (0)	0.230
Post PD-1 inhibitors	4 (22.2)	1 (6.3)	0.340
TACE/surgical resection	2 (11.1)	0 (0)	0.487
Clinical trial	1 (5.6)	0 (0)	1.000
TKI	1 (5.6)	1 (6.3)	1.000

*PD-1* programmed cell death protein-1, *TACE* transcatheter arterial chemoembolization, *TKI* tyrosine kinase inhibitor

^a^Data are reported as *n* (%)

At the end of the follow-up period, 11 patients (61.1%) with vascular responsiveness and 15 patients (93.8%) without vascular responsiveness died (*p* = 0.043). At the end of study period, three patients (16.7%) with vascular responsiveness were still in the treatment with PD-1 inhibitors. Four vascular responders (22.2%) underwent post-ICI treatments, including 1 with local treatment with TACE; 1 with surgical resection for primary hepatic tumor; 1 enrolled into a clinical trial; and 1 TKI with lenvatinib. Among the patients without tumor thrombus who achieved overall objective response, three patients were still undergoing treatment with PD-1 inhibitors, and one patient had to undergo further curative resection for primary hepatic tumor (Supplementary Table 2).

**Fig. 2:**
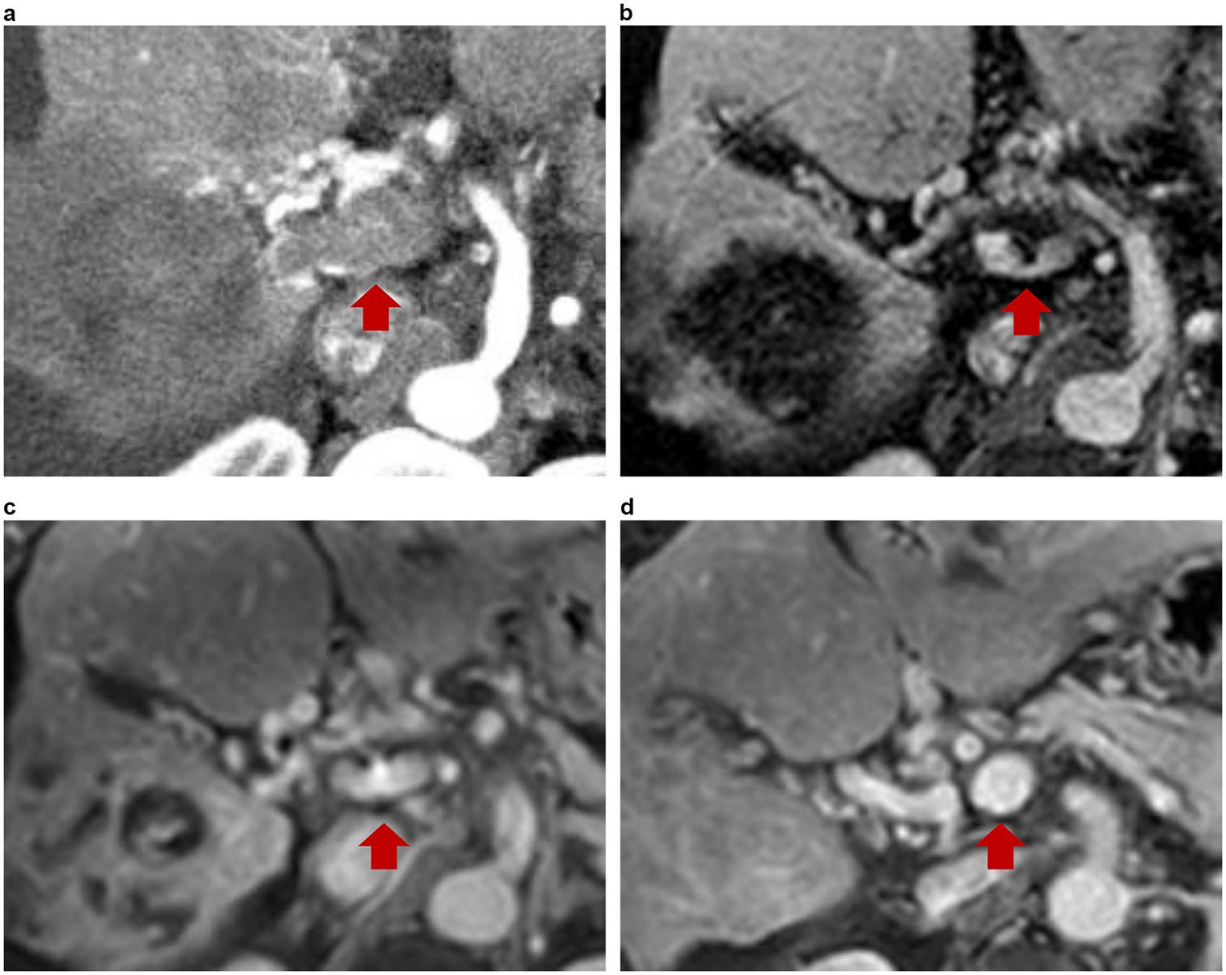
Serial magnetic resonance imaging of a 65-year-old male with hepatocellular carcinoma (HCC) with main portal vein tumor thrombus (PVTT) treated with a PD-1 inhibitor (**a**) before treatment, the thrombus was nodular, expanded, and enhanced in the main portal vein (arrow in **a**). Scans taken 2 (**b**), 5 (**c**), and 13 (**d**) months after treatment, showing marked PVTT regression

As illustrated in Fig. 2, the follow-up images of patients with main PVTT indicated complete disappearance of tumor thrombi after PD-1 inhibitor treatment.

### Survival analysis and predictors of survival

The median follow-up period for the overall cohort was 7.2 months (interquartile range 3.3–11.6 months). The median OS was 12.1 [95% confidence interval (CI) 7.4–not estimable] months for patients without vascular thrombi and 8.9 (95% CI 3.2–12.6) months for those with tumor thrombi (*p* = 0.020). The median PFS was 3.3 (95% CI 2.4–6.0) months for patients without vascular thrombi and 3.8 (95% CI 2.5–6.9) months for those with tumor thrombi (*p* = 0.787). The median OS was not reached for patients with objective response among those with and without tumor thrombi (Supplementary Fig. 2).

**Fig. 3:**
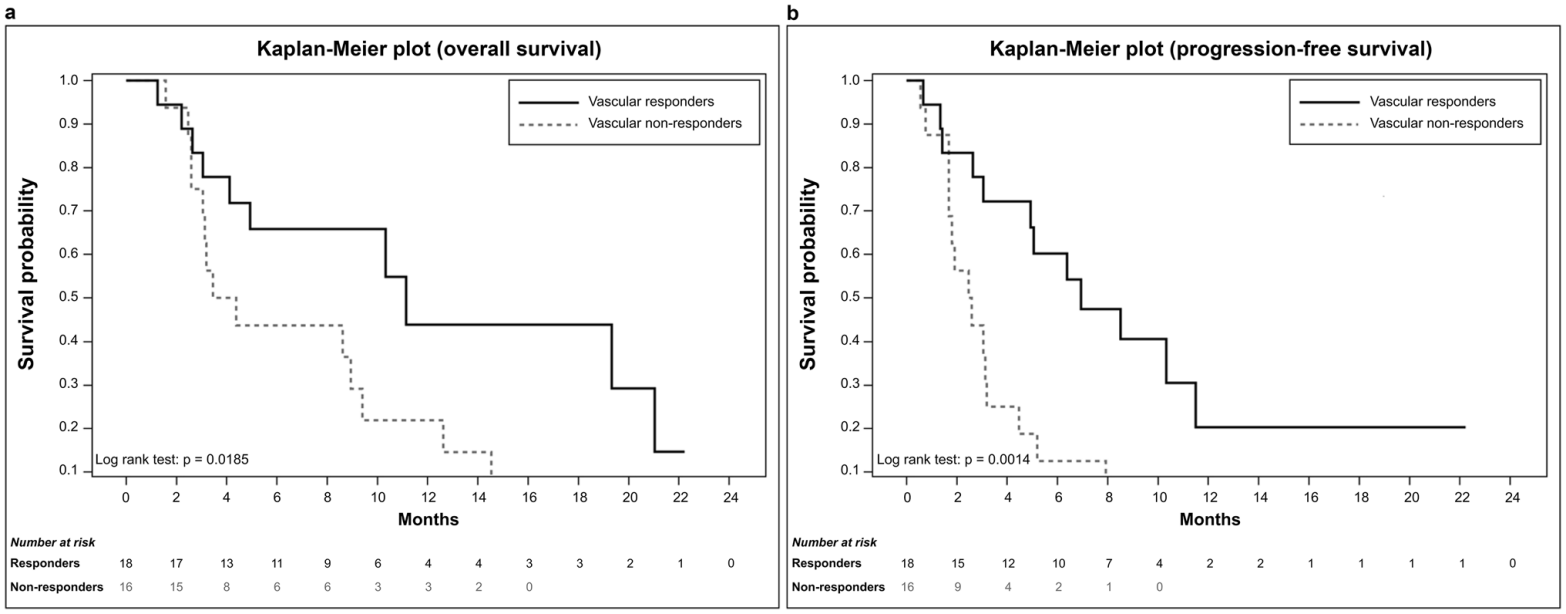
Overall survival (**a**) and progression-free survival (**b**) according to the response of vascular metastases. Responders had significantly longer survival than did non-responders

The median OS for patients exhibiting a vascular response was 11.1 (95% CI 4.1–21.0) months and was significantly longer than that of patients without a vascular response (3.9 months; 95% CI 2.6–9.4 months; *p* = 0.018). The PFS was 6.9 (95% CI 3.1–11.5) months and 2.5 (95% CI 1.7–3.2) months for vascular responders and non-responders, respectively (*p* = 0.001), as presented in Fig. 3.

Univariate analysis of OS implied that performance status, advanced CLIP stage, Child–Pugh class, previous surgical resection, and vascular response were prognostic factors for OS (Online Supplementary Table 3). In regard to multivariate analysis, the vascular response appeared to be a significant prognostic indicator associated with OS.

### Safety

In the present study, the overall incidence of treatment-related adverse events for patients with and without tumor thrombi were 32.4% and 47.0%, respectively (*p* = 0.322). The most commonly reported adverse events for patients with tumor thrombi were rash (*n* = 6; 17.6%), pneumonitis (*n* = 2; 5.9%), hepatitis (*n* = 2; 5.9%), and fatigue (*n* = 1; 2.9%) (Table 3). Treatment-related serious adverse events were reported in two patients (5.9%) with acute respiratory distress syndrome and one (2.9%) with hepatitis.

**Table 3:** Incidence of adverse events in patients with tumor thrombi

Adverse event, *n* (%)	Any grade	Grade 3	Grade 4
Overall incidence	11 (32.4)	1 (2.9)	2 (5.9)
Rash	6 (17.6)	0 (0)	0 (0)
Hepatitis	2 (5.9)	1 (2.9)	0 (0)
Pneumonitis	2 (5.9)	0 (0)	2 (5.9)
Fatigue	1 (2.9)	0 (0)	0 (0)

## Discussion

The present study provides valuable information on the response of tumor thrombi and clinical outcomes after PD-1 inhibitors in patients with advanced HCC and MVI. As a result, the overall ORR and survival of those with objective response were comparable between patients with and without tumor thrombi. Whereas the response rate of vascular tumor thrombosis was 52.9%, and the responders appear to have a more survival benefit than non-responders. Furthermore, the MVI responsiveness closely correlated with the maintenance of optimal liver function and a lower occurrence of distal metastases. Two patients who exhibited a vascular response underwent surgery and TACE without further systemic therapy. Our findings are in agreement with a report which addressed a complete response of IVCT to ICIs in advanced-stage renal cell carcinoma and proposed the response of vascular thrombi to ICIs being stronger in a high T-cell inflamed tumor microenvironment [22]. These findings indicate that ICIs markedly decrease or stabilize tumor thrombus volume, and this response may be affected by the diversity of tumor microenvironments [13, 14, 23]. In addition, the regression of vascular metastases may preserve organ function and prevent distant metastasis, thus offering further curative treatment either alone or in combination with other modalities for non-responding organs. Hence, in managing these clinically challenging cases, immunotherapy should be considered as a first-priority in an effort to avoid further delays for the HCC patients with MVI.

Vascular invasion is associated with poorer OS in HCC patients. The management of HCC with vascular invasion remains quite challenging, and its therapeutic options are very limited [3, 16]. Immunotherapy with PD-1 inhibitors exhibits very promising anti-cancer effects and has been approved by the FDA as a second-line agent for the advanced HCC [10]. However, the therapeutic benefit of ICIs for advanced HCC patients with vascular tumor thrombosis still remains unclear. Clinical trials have generally excluded patients with main PVTT or IVCT; thus, it’s rare to provide evidence regarding the efficacy of immunotherapy for these patients. To the best of our knowledge, this study was the first to investigate the outcomes of PD‐1 targeted immunotherapy for the HCC patients with advanced MVI using data acquired in a clinical setting. In this real-world analysis, an ORR of 17.6% and an OS of 8.9 months were observed with most toxicities manageable. The ORR was similar between patients with and without tumor thrombi treated with PD-1 inhibitors. Furthermore, survival of those with objective response did not significantly differ between patients with and without thrombi. Therefore, immunotherapy with PD-1 inhibitors may serve as a feasible treatment option in HCC with tumor thrombi and may be considered as a potential alternative therapy for clinically difficult cases. Future prospective studies are needed to verify the results of the current study and the effects of immunotherapy administered in combination with other strategies in HCC with MVI.

Although sorafenib is generally accepted as a standard treatment approach in advanced HCC with MVI, the overall ORR of 10.0% is relatively low and survival is prolonged only by 3.1 months [6]. Radiation, arterial infusion of chemotherapy, and transarterial chemoembolization are the current options for eliminating tumor thrombi; however, the indications for these approaches are often limited due to the extent of the lesion or impaired liver function [16]. In the present study, the ORRT of 52.9% reflects the remarkable regression of tumor thrombi following PD-1 inhibitor treatment given that there is no definite cure for HCC with MVI. The vascular responders closely correlated with the maintenance of optimal liver function, with 11% of the patients converting to local-regional treatment. These findings support the clinically important implication that immunotherapy with PD-1 inhibitors may contribute to the effective control of tumor thrombi, preserve liver function, and provide an opportunity to receive further treatment. Moreover, the effective vascular response to PD-1 inhibitors in patients with tumor thrombi has important clinical implications regarding patient survival. Hence, the present study is the first to illustrate that immunotherapy with PD-1 inhibitors can achieve a good response rate of tumor thrombi and favorable survival outcomes. However, still a limited proportion of patients with advanced HCC and MVI exhibited a favorable outcome after their immunotherapy. Obviously, further research is urgently needed to predict good responders to personalized therapy as well as the results of clinical trials employing immunotherapy in earlier stage HCC.

As observed in this study, no difference exists in efficacy between the use of PD-1 inhibitors as a first‐line and second/third‐line therapy. These findings support the notion that ICIs may be an effective treatment for the HCC patients with advanced MVI and that ICI use should be considered as an alternative or rescue option for these patients. Besides, even in Child–Pugh class B patients, immunotherapy led to a disease stabilization in about half of the patients (50% overall DCR), similar to those of previous studies [11, 15]. Such findings suggest that even for the patients with more advanced liver function impairment, treatment with ICI could still be beneficial and may provide an alternative treatment strategy for the HCC patients with impaired liver functional reserve.

Combination therapy has been reported in recent clinical trials for its potential synergic effect and superior survival benefit in the advanced HCC [24]. In our cohort, combined therapy with PD-1 inhibitors and TKIs was not identified as a prognostic factor for OS, in agreement with previous studies [25, 26]. The patients in our study were heterogeneous with respect to TKI regimen and previous systemic therapy. To evaluate the effect of combination therapy on tumor thrombi, it’s definitely needed to conduct prospective and large-scale research in the near future.

A previous study reported preserved liver function (Child–Pugh A) as an independent predictor to improve OS in patients receiving PD-1 inhibitor treatment [26]. However, in this study, it did not independently predict overall mortality by multivariate analysis; this may be attributable to the limited number of cases and high-risk study population. Therefore, future large cohort-based, long-term follow-up studies are required to determine the possible predictive factors that influence overall mortality. Moreover, the absence of a vascular response was the only independent indicator of decreased OS. These results were similar to those of studies conducted for HCC with vascular invasion treated with radiotherapy in which OS was significantly affected by vascular response [27, 28]. Based on these findings, a treatment modality that can produce a good response rate to tumor thrombi should be considered as an initial strategy in HCC treatment with MVI.

Concerning the limitation of this study, several aspects need to be addressed. First, the small cohort size and retrospective nature of the analysis may not support the implication in a more persuasive way. However, the data presented in this study together with a previous report indicate a potential of immunotherapy for further clinical application and research in cancers with tumor thrombi [22]. Future prospective and large-scale studies are needed to verify the results of the current study. Second, the vascular response was assessed by measuring the decrease in thrombus size; thus, the assessment may be inadequate in the case of vascular responders in whom thrombus growth was arrested without a decrease in size [20, 28]. Alternative assessment methods not based on thrombus shrinkage are needed for a more accurate measurement of the thrombus response to ICIs.

In conclusion, to the contrary of the past cases, the use of PD-1 inhibitors results in tumor thrombus regression and the increased patient survival according to this clinically challenging cohort of advanced HCC patients. Thus, ICIs may serve as an effective therapeutic agent for treating malignant thrombi, potential prevention of derangement in liver function, and elimination of distant metastasis, thus offering a strategy for preventing progression into advanced stage of cancer. Future investigations are needed to reveal the mechanism underlying the antithrombotic effect of ICIs and to identify predictive biomarkers for determining the efficacy of ICIs therapy for the patients with HCC.

### Supplementary Information

Below is the link to the electronic supplementary material.

Supplementary file1 (PDF 199 KB)

Supplementary file2 (PDF 144 KB)

Supplementary file3 (PDF 195 KB)

Supplementary file4 (PDF 118 KB)

Supplementary file5 (PDF 31 KB)

Supplementary file6 (PDF 146 KB)

## Abbreviations

BCLC: Barcelona clinic liver cancer
CR: Complete remission
CT: Computed tomography
DCR: Disease control rate
DCRT: Disease control rate of tumor thrombi
HCC: Hepatocellular carcinoma
ICI: Immune checkpoint inhibitors
IVC: Inferior vena cava
IVCT: Inferior vena cava thrombus
MRI: Magnetic resonance imaging
ORR: Objective response rate
ORRT: Objective response rate of tumor thrombi
OS: Overall survival
PD: Progressive disease
PFS: Progression-free survival
PR: Partial response
PVTT: Portal vein tumor thrombus
RECIST: Response evaluation criteria in solid tumors
SD: Stable disease
TKI: Tyrosine kinase inhibitor
