# Supplementary material for: Real-world outcome of immune checkpoint inhibitors for advanced hepatocellular carcinoma with macrovascular tumor thrombosis
Source: Cancer Immunol Immunother. 2021 Jan 6;70(7):1929–37. doi: 10.1007/s00262-020-02845-9 (PMC8195886; doi:10.1007/s00262-020-02845-9)
Supplement: Supplementary file 1 — Supplementary file1 (PDF 199 KB) [file 262_2020_2845_MOESM1_ESM.pdf]

Supplementary Table 1. Baseline characteristics of all patients

Characteristic	Without thrombi (n = 34)	With thrombi (n = 34)	*p*
**Sex, n (%)**			
Female	7 (20.6)	6 (17.7)	1.000
Male	27 (79.4)	28 (82.4)	
**Median age (IQR), years**	61.0 (52.0, 70.0)	60.5 (52.8, 65.3)	0.868
< 55	9 (26.5)	9 (26.5)	
≥ 55	25 (73.5)	25 (73.5)	
**ECOG, n (%)**			
0	26 (76.5)	20 (58.8)	0.136
1	8 (23.5)	10 (29.4)	
2	0 (0.0)	3 (8.8)	
3	0 (0.0)	1 (2.9)	
**Alpha-fetoprotein, ng/mL**†			
< 400	20 (60.6)	19 (61.3)	1.000
≥ 400	13 (39.4)	12 (38.7)	
**Etiology of liver disease, n (%)**			
No liver disease	5 (14.7)	4 (11.8)	1.000

Liver disease present	29 (85.3)	30 (88.2)	1.000
Chronic hepatitis B	28 (96.6)	23 (76.7)	
Chronic hepatitis C	0 (0.0)	6 (20.0)	
Alcoholic hepatitis	1 (3.4)	1 (3.3)	
**Child–Pugh stage, n (%)**			
A	29 (85.3)	22 (64.7)	0.091
B	5 (14.7)	12 (35.3)	
**CLIP, n (%)**			
0-1	23 (67.6)	8 (23.5)	0.001
2-5	11 (32.4)	26 (76.5)	
**Distant metastases, n (%)**			
No	3 (8.8)	16 (47.1)	0.001
Yes	30 (88.2)	18 (52.9)	
Lung	20 (66.7)	11 (61.1)	0.938
Bone	8 (26.7)	3 (16.7)	0.499
Lymph node	14 (46.7)	10 (55.6)	0.766
Other[‡]	10 (33.3)	7 (38.9)	0.938
**Thrombus location, n (%)**			
IVC		6 (17.6)	

IVC + 1st branch PV		4 (11.8)	
Main PV + bilateral 1st branch PV		14 (41.2)	
Main PV + 1st branch PV		1 (2.9)	
Main PV		1 (2.9)	
Bilateral 1st branch PV		1 (2.9)	
1st branch PV		7 (20.6)	
**Previous treatment, n (%)**			
No	2 (5.9)	7 (20.6)	0.150
Yes	32 (94.1)	27 (79.4)	
Surgical resection	17 (53.1)	12 (44.4)	0.687
TACE	22 (68.8)	13 (48.2)	0.181
RFA/PEI	11 (34.4)	8 (29.6)	0.913
HAIC	8 (25.0)	12 (44.4)	0.195
Previous sorafenib	27 (84.4)	21 (77.8)	0.755
Previous lenvatinib	1 (3.1)	1 (3.7)	1.000
**PD-1 inhibitors as systemic therapy, n (%)**			
First-line	5 (14.7)	11 (32.4)	0.249
Second-line	27 (79.4)	22 (64.7)	
Third-line	2 (5.9)	1 (2.9)	

**PD-1 inhibitors combined with TKIs, n (%)**			
No	13 (38.2)	13 (38.2)	1.000
Yes	21 (61.8)	21 (61.8)	
Sorafenib	11 (52.4)	16 (76.2)	0.198
Regorafenib	2 (9.5)	1 (4.8)	1.000
Lenvatinib	8 (38.1)	4 (19.0)	0.306

†With tumor thrombi: three missing values; without tumor thrombi: one missing value

‡With tumor thrombi: 3, 3, and 1 patient with peritoneal metastases, adrenal involvement, and diaphragm invasion, respectively; without tumor thrombi: 6, 3, and 1 patient with peritoneal metastases, adrenal involvement, and diaphragm invasion, respectively

ECOG, Eastern Cooperative Oncology Group performance status; CLIP, Cancer of the Liver Italian Program Scoring System; BCLC, Barcelona Clinic Liver Cancer; IVC, inferior vena cava; PV, portal vein; TACE, transcatheter arterial chemoembolization; RFA, radiofrequency ablation; PEI, percutaneous ethanol injection; HAIC, hepatic arterial infusion chemotherapy; PD-1, programmed cell death protein-1; TKI, tyrosine kinase inhibitor
