# Supplementary material for: Real-world outcome of immune checkpoint inhibitors for advanced hepatocellular carcinoma with macrovascular tumor thrombosis
Source: Cancer Immunol Immunother. 2021 Jan 6;70(7):1929–37. doi: 10.1007/s00262-020-02845-9 (PMC8195886; doi:10.1007/s00262-020-02845-9)
Supplement: Supplementary file 2 — Supplementary file2 (PDF 144 KB) [file 262_2020_2845_MOESM2_ESM.pdf]

Supplementary Table 2. Analysis of factors associated with overall tumor response in patients without tumor thrombi

	**Without tumor thrombi** (n = 34)		
	Overall CR/PR[†‡] (n = 4)	Overall SD/PD[†‡] (n = 30)	*p*
**Child–Pugh score/class elevation**	0 (0)	7 (23.3)	0.559
**New distant metastasis**	0 (0)	2 (6.7)	1.000
**Death**	0 (0)	16 (53.3)	0.105
**Ongoing ICI treatment**	3 (75.0)	3 (10.0)	0.012
**Post PD-1 inhibitors**	1 (25.0)	11 (36.7)	1.000
TACE/surgical resection	1 (25.0)	0 (0)	0.118
Clinical trial	0 (0)	2 (6.7)	1.000
TKI	0 (0)	6 (20.0)	1.000
Systemic chemotherapy	0 (0)	2 (6.7)	1.000
HAIC	0 (0)	1 (3.3)	1.000

[†]Data are reported as n (%).

[‡]Response was evaluated using the RECIST.

PD-1, programmed cell death protein-1; TACE, transcatheter arterial chemoembolization; TKI, tyrosine kinase inhibitor; HAIC, hepatic arterial infusion chemotherapy; CR, complete response; PR, partial response; SD, stable disease; PD, progressive disease
