# Supplementary material for: Real-world outcome of immune checkpoint inhibitors for advanced hepatocellular carcinoma with macrovascular tumor thrombosis
Source: Cancer Immunol Immunother. 2021 Jan 6;70(7):1929–37. doi: 10.1007/s00262-020-02845-9 (PMC8195886; doi:10.1007/s00262-020-02845-9)
Supplement: Supplementary file 3 — Supplementary file3 (PDF 195 KB) [file 262_2020_2845_MOESM3_ESM.pdf]

Supplementary Table 3. Predictive factors influencing overall mortality

	Crude HR (95% CI)	*p*	Adjusted HR† (95% CI)	*p*
**Sex**				
Female	Ref.			
Male	0.78 (0.28–2.12)	0.620		
**Age (y)**				
<55	Ref.			
≥55	0.88 (0.36–2.12)	0.768		
**ECOG**				
0	Ref.		Ref.	
≥1	3.08 (1.38–6.87)	0.006	1.94 (0.71–5.26)	0.194
**Alpha-fetoprotein (ng/mL)**				
<400	Ref.			

≥400	2.55 (0.99–6.56)	0.052		
**Etiology of chronic liver disease**				
No liver disease	Ref.			
Liver disease present	0.75 (0.25–2.24)	0.606		
**CLIP**				
0–1	Ref.		Ref.	
2–5	3.79 (1.31–10.94)	0.014	2.46 (0.61–10.00)	0.207
**Child–Pugh stage**				
A	Ref.		Ref.	
B	2.63 (1.11–6.24)	0.028	1.40 (0.51–3.83)	0.515
**Distant metastases**				
No	Ref.			
Yes	1.00 (0.44–2.27)	0.994		
**Previous treatment**				
No	Ref.			

Yes	0.94 (0.34–2.55)	0.897		
Surgical resection	0.38 (0.15–0.96)	0.041	0.57 (0.20–1.64)	0.295
TACE	0.77 (0.33–1.80)	0.551		
RFA/PEI	0.71 (0.26–1.93)	0.501		
TKIs	0.58 (0.25–1.34)	0.201		
**PD-1 inhibitors combined with TKIs**				
No	Ref.			
Yes	0.74 (0.31–1.74)	0.488		
**Vascular response**				
No	Ref.		Ref.	
Yes	0.37 (0.16–0.88)	0.024	0.30 (0.10–0.84)	0.022

† Cox proportional hazards models

Vascular response was defined as the total number of patients with complete response and partial response of the tumor thrombi.

HR, hazard ratio; CI, confidence interval; ECOG, Eastern Cooperative Oncology Group performance status; CLIP, Cancer of the Liver Italian Program Scoring System; TACE, transcatheter arterial chemoembolization; RFA, radiofrequency ablation; PEI, percutaneous ethanol injection;

TKI, tyrosine kinase inhibitor; PD-1, programmed cell death protein-1; Ref., reference level
