# Supplementary material for: Real-world outcome of immune checkpoint inhibitors for advanced hepatocellular carcinoma with macrovascular tumor thrombosis
Source: Cancer Immunol Immunother. 2021 Jan 6;70(7):1929–37. doi: 10.1007/s00262-020-02845-9 (PMC8195886; doi:10.1007/s00262-020-02845-9)
Supplement: Supplementary file 4 — Supplementary file4 (PDF 118 KB) [file 262_2020_2845_MOESM4_ESM.pdf]

Supplementary Figure Legends

Supplementary Figure S1. Dynamic changes in serum total bilirubin levels according to the response of vascular metastases to PD-1 inhibitor therapy. Note the trend toward elevated total bilirubin levels in vascular non-responders.

Supplementary Figure S2. Overall survival stratified by tumor thrombi in (a) patients with objective response and (b) in those without objective response after treatment with PD-1 inhibitors. Survival is comparable between patients with tumor thrombi who achieved objective response and those without tumor thrombi who achieved objective response (p = 0.371).
