# Supplementary figures and images for: Real-world outcome of immune checkpoint inhibitors for advanced hepatocellular carcinoma with macrovascular tumor thrombosis
Source: Cancer Immunol Immunother. 2021 Jan 6;70(7):1929–37. doi: 10.1007/s00262-020-02845-9 (PMC8195886; doi:10.1007/s00262-020-02845-9)

Supplementary Figure 1

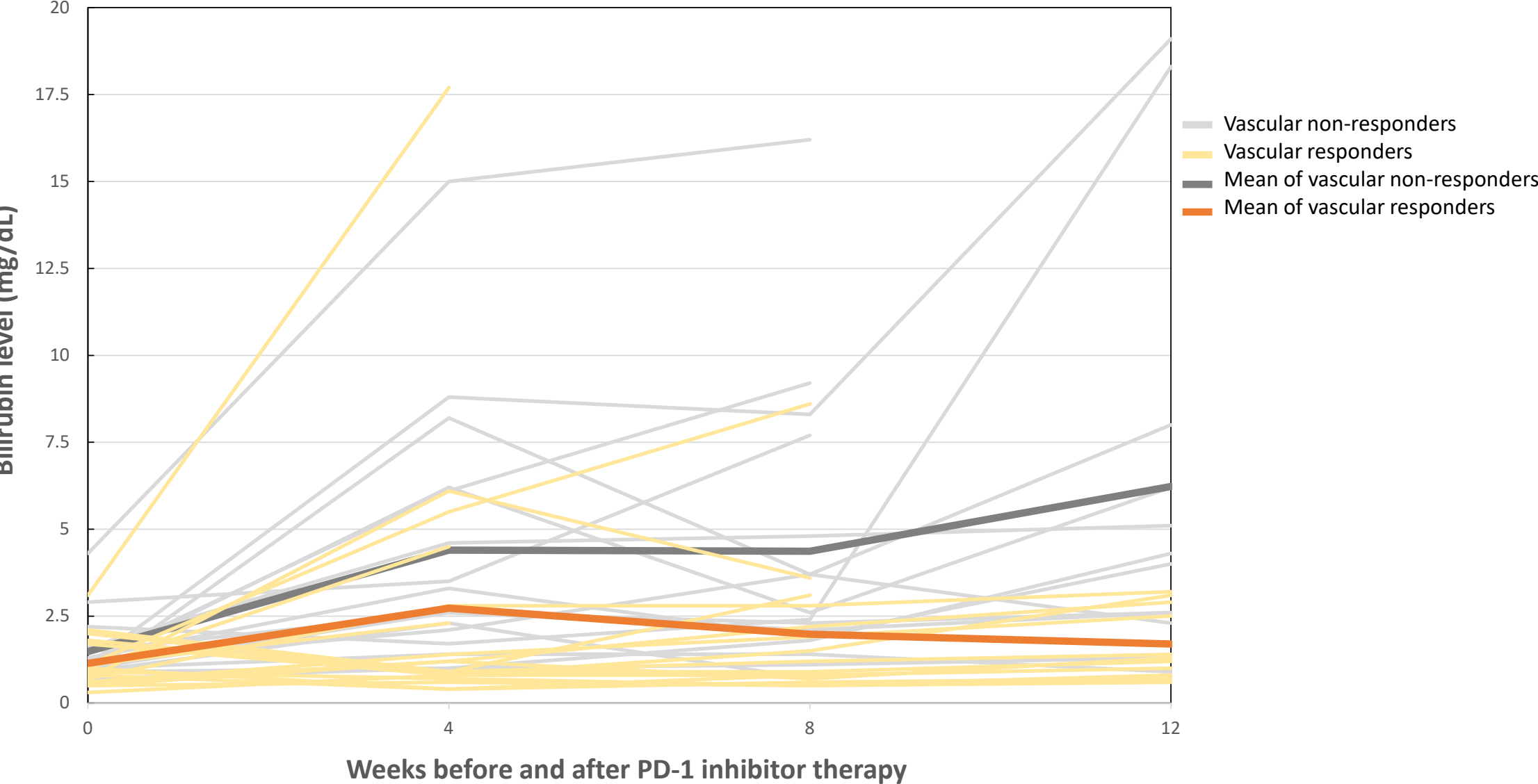

Supplement: Supplementary file 5 — Supplementary file5 (PDF 31 KB) [file 262_2020_2845_MOESM5_ESM.pdf]

# Supplementary Figure 2

a

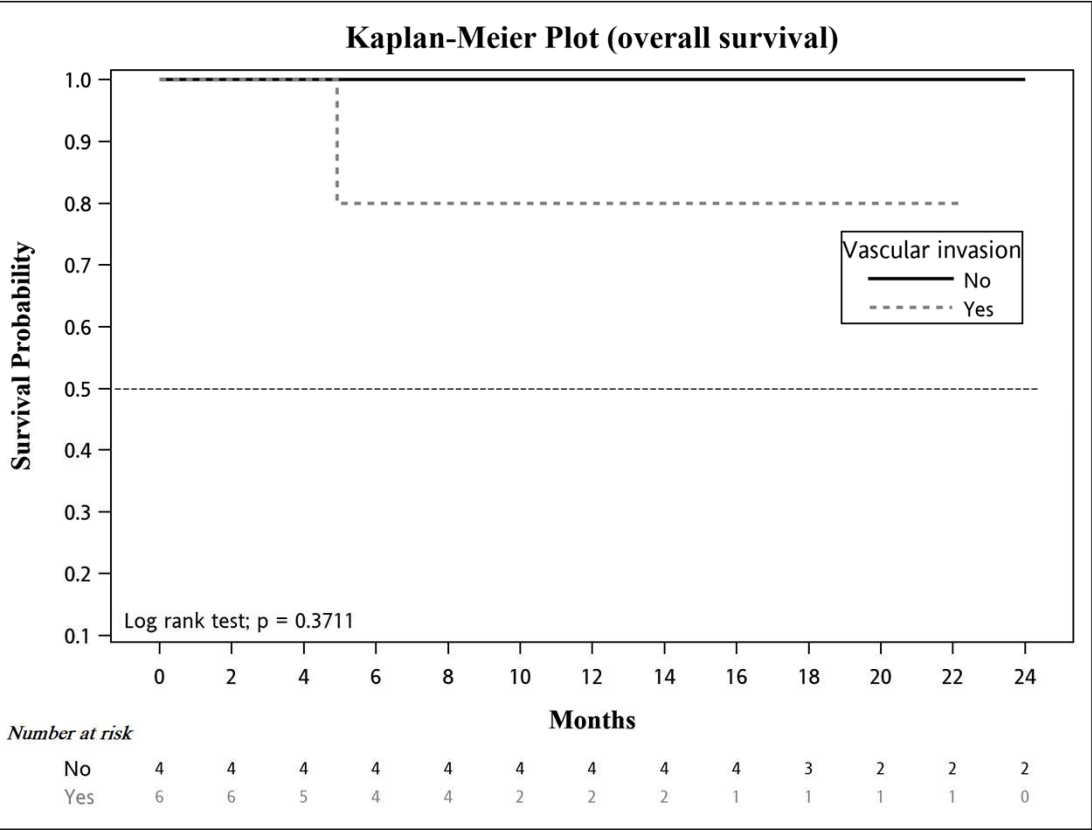

b

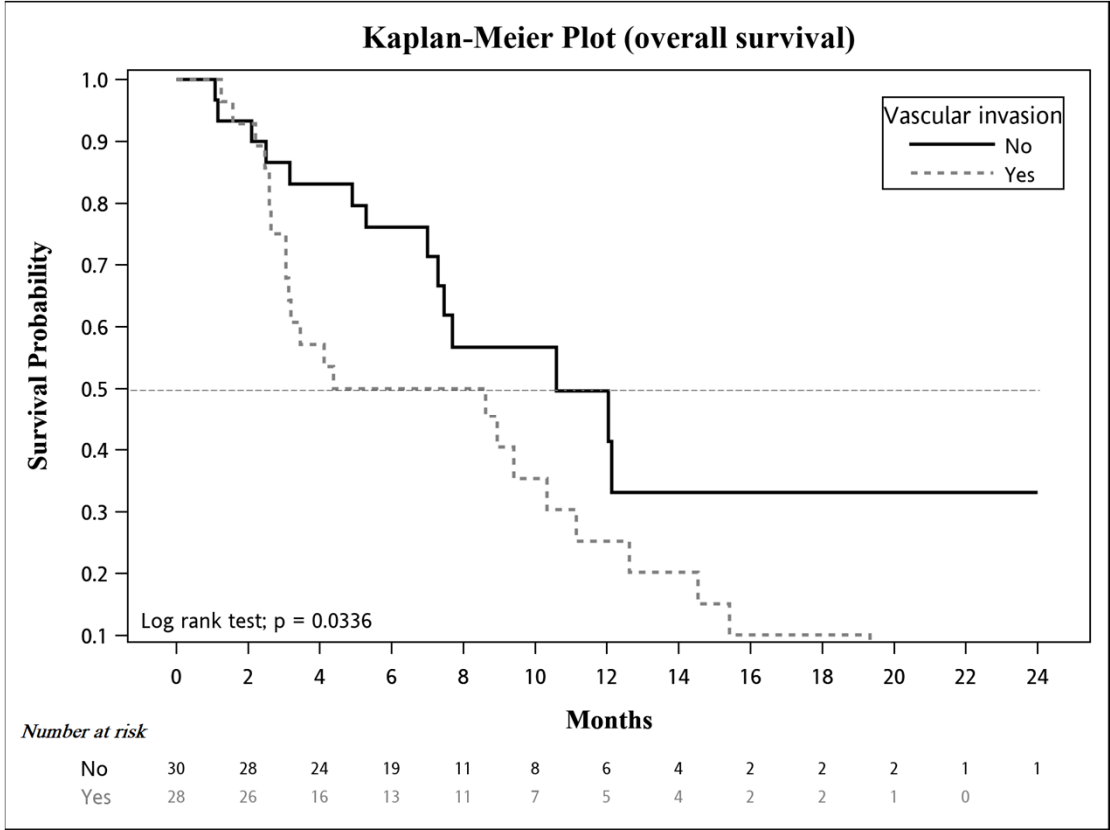

Supplement: Supplementary file 6 — Supplementary file6 (PDF 146 KB) [file 262_2020_2845_MOESM6_ESM.pdf]
